# Antimicrobial Activity of UV-Induced Phenylamides from Rice Leaves

**DOI:** 10.3390/molecules191118139

**Published:** 2014-11-06

**Authors:** Hye Lin Park, Youngchul Yoo, Tae-Ryong Hahn, Seong Hee Bhoo, Sang-Won Lee, Man-Ho Cho

**Affiliations:** 1 Graduate School of Biotechnology, Kyung Hee University, Yongin 446-70, Korea; E-Mails: hlpark@khu.ac.kr (H.L.P.); trhahn@khu.ac.kr (T.-R.H.); shbhoo@khu.ac.kr (S.H.B.); 2 Department of Plant Molecular Systems Biotechnology & Crop Biotech Institute, Kyung Hee University, Yongin 446-70, Korea; E-Mail: yooyc@khu.ac.kr

**Keywords:** rice, UV treatment, phenylamide, phytoalexin, *N*-*trans*-cinnamoyltryptamine, *N*-*p*-coumaroylserotonin, *N*-*trans*-cinnamoyltyramine, *N*-benzoyltryptamine, antimicrobial activity

## Abstract

Rice produces a wide array of phytoalexins in response to pathogen attacks and UV-irradiation. Except for the flavonoid sakuranetin, most phytoalexins identified in rice are diterpenoid compounds. Analysis of phenolic-enriched fractions from UV-treated rice leaves showed that several phenolic compounds in addition to sakuranetin accumulated remarkably in rice leaves. We isolated two compounds from UV-treated rice leaves using silica gel column chromatography and preparative HPLC. The isolated phenolic compounds were identified as phenylamide compounds: *N*-*trans*-cinnamoyltryptamine and *N*-*p*-coumaroylserotonin. Expression analysis of biosynthetic genes demonstrated that genes for arylamine biosynthesis were upregulated by UV irradiation. This result suggested that phenylamide biosynthetic pathways are activated in rice leaves by UV treatment. To unravel the role of UV-induced phenylamides as phytoalexins, we examined their antimicrobial activity against rice fungal and bacterial pathogens. *N*-*trans*-Cinnamoyltryptamine inhibited the growth of rice brown spot fungus (*Bipolaris oryzae*). In addition to the known antifungal activity to the blast fungus, sakuranetin had antimicrobial activity toward *B. oryzae* and *Rhizoctonia solani* (rice sheath blight fungus). UV-induced phenylamides and sakuranetin also had antimicrobial activity against rice bacterial pathogens for grain rot (*Burkholderia glumae*), blight (*Xanthomonas oryzae* pv. *oryzae*) and leaf streak (*X. oryzae* pv. *oryzicola*) diseases. These findings suggested that the UV-induced phenylamides in rice are phytoalexins against a diverse array of pathogens.

## 1. Introduction

Plants produce antimicrobial secondary metabolites, called phytoalexins, as a defense against pathogens. Diterpenoid and flavonoid phytoalexins are produced in rice in response to pathogens and environmental stress [1,2,3,4,5,6,7,8,9,10,11,12]. Momilactones A and B isolated from rice seed husks have antifungal activity against *Magnaporthe grisea*, the fungal pathogen that cause rice blast disease [1,13]. Since the finding of momilactones A and B, many phytoalexins have been identified from rice. Oryzalexins A–D, phytocassanes A–D and *ent*-10-oxodepressin are found to be induced by *M. grisea* infection in rice [3,4,9,12]. Phytocassane E was identified in rice infected with *Rhizoctonia* *solani* [10]. Production of most phytoalexins, including the momilactones and oryzalexins, is induced by UV-irradiation [2,5,7,8]. The production of diterpenoid phytoalexins is also induced by treatment with jasmonic acid, CuCl_2_ or chitin elicitor [14,15,16]. Sakuranetin was isolated from UV-treated rice leaves and has antifungal activity against *M. grisea* [6]. Most rice phytoalexins, except sakuranetin, are structurally related and grouped as diterpenoids [17,18,19]. The flavonoid sakuranetin is a phenolic phytoalexin.

Phenylamides are the conjugated form of phenolic acids and amines and are widely distributed throughout the plant kingdom [20]. They function in growth, development and floral initiation [20,21]. Accumulating evidence shows that phenylamides participate in defense against biotic and abiotic stress in some plant species [22,23,24,25,26,27,28,29]. Production of phenylamide compounds, such as feruloyltryptamine and *p*-coumaroylserotonin, in rice leaves is induced by the infection by the rice brown spot fungus *Bipolaris oryzae* [30]. However, antimicrobial activity of phenylamides against rice pathogens has not been reported yet.

We previously found that in addition to sakuranetin, several phenolic compounds accumulate in UV-treated rice leaves [31,32]. The UV-induced phenolic compounds include *N*-*trans*-cinnamoyltyramine and *N*-benzoyltryptamine [32]. In this study, we isolated two additional phenolic compounds from UV-treated rice leaves that were also identified as phenylamides. These findings indicated that UV radiation substantially induced the production of phenylamides. Gene expression analysis also showed the induction of arylamine and phenolic acid biosynthetic pathways in UV-treated rice leaves. The UV-induced phenylamides were tested as phytoalexins similar to sakuranetin and other diterpenoid phytoalexins by examining their antimicrobial activity against rice pathogens.

## 2. Results and Discussion

### 2.1. Phenylamides Accumulate in UV-Treated Rice Leaves

**Figure 1:**
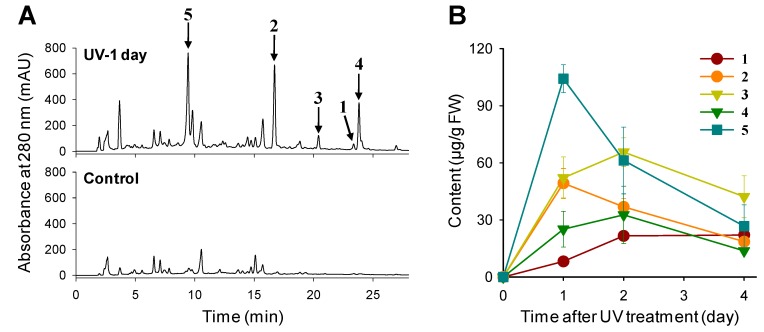
UV-Induced accumulation of phenolic compounds in rice leaves. (**A**) HPLC-UV (280 nm) analysis of phenolic-enriched fractions extracted from rice leaves collected at 1 day after UV treatment and untreated control. Phenolic compounds induced in UV-treated rice leaves were designated as **1**–**5**. (**B**) Contents of phenolic compounds in rice leaves collected at 1, 2 and 4 days after UV treatment. FW; fresh weight.

Synthesis of most rice phytoalexins including sakuranetin is stimulated by UV irradiation [2,5,6,7,8,31]. In a previous study, we analyzed phenolic-enriched extracts from UV-treated rice leaves and found that, in addition to sakuranetin (**1**), accumulation of four potential phenolic compounds **2**‒**5** increased remarkably after UV irradiation (Figure 1A). These phenolic compounds were rapidly accumulated in rice leaves after UV treatment (Figure 1B). Among the UV-induced phenolic compounds, compounds **2** and **3** had been previously identified as *N*-*trans*-cinnamoyltyramine and *N*-benzoyltryptamine, respectively, suggesting that UV-irradiation induces the synthesis of known phytoalexins and phenylamides in rice leaves [32]. 

**Figure 2:**
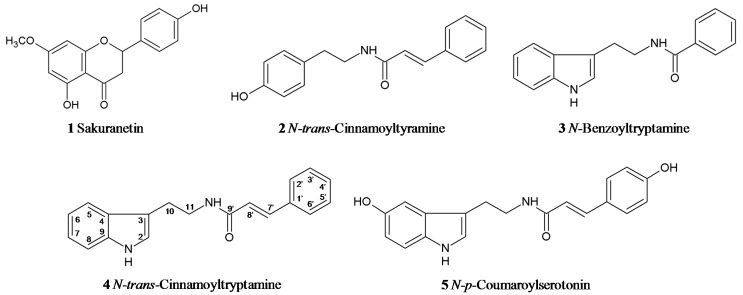
Structure of phenylamides isolated from UV-treated rice leaves. Compounds **4** and **5** were isolated from UV-treated rice leaves and identified as *N*-*trans*-cinnamoyltryptamine and *N*-*p*-coumaroylserotonin using NMR and MS analysis, respectively. Compounds **2** and **3** were previously identified as *N*-*trans*-cinnamoyltyramine and *N*-benzoyltryptamine, respectively [32].

We isolated compounds **4** and **5** from rice leaves collected 1 day after UV treatment using silica gel column chromatography and preparative high performance liquid chromatography (HPLC). The isolated compounds were identified using NMR spectroscopies and positive electron spray ionization mass spectrometry (ESI MS). Typical proton signals for cinnamoyl and tryptamyl moieties were observed in the ^1^H-NMR spectrum of compound **4**. In a heteronuclear multiple bond correlation (HMBC) spectrum, a methine proton signal at δ 3.60 (H-11) was correlated with a carbonyl carbon signal in the cinnamoyl moiety at δ 168.8 (C-9'). These findings indicated that a tryptamyl moiety connected to the cinnamoyl moiety via an amino bond. The pseudomolecular ion peak of compound **4** was detected at *m/z* 291.3 [M+H]^+^ in positive ESI MS. These results identified compound **4** as *N*-*trans*-cinnamoyltryptamine (Figure 2). 

^1^H and ^1^H-^1^H correlation spectra suggested that compound **5** had a similar structure to compound **4**. Compound **5** lacked two proton signals for H-6 and H-4' of compound **4**. Carbon signals for C-6 and C-4' in compound **5** were observed at δ 151.3 and δ 160.7, respectively, suggesting hydroxylation of these positions. Thus, compound **5** appeared to be composed of serotonin and *p*-coumaroyl moieties. Correlation between the methine proton signal in the serotonin moiety at δ 3.56 (H-11) and the carbonyl carbon signal in the *p*-coumaroyl moiety at δ 169.4 (C-9') in the HMBC spectrum indicated that serotonin and *p*-coumaroyl moieties are connected via an amino bond. NMR data of compound **5** were consistent with data of *N*-*p*-coumaroylserotonin, which was identified previously from safflower seeds [33]. The pseudomolecular ion peak of compound **5** observed at *m/z* 323.1 [M+H]^+^ in the positive ESI MS was consistent with *N*-*p*-coumaroylserotonin. These data identified compound **5** as *N*-*p*-coumaroylserotonin (Figure 2). ^1^H- and ^13^C-NMR data of compounds **4** and **5** are given in Table S1. Besides **1**–**5**, a putative phenolic compound at retention time 3.7 min was induced by UV treatment (Figure 1A). This compound is needed to be further isolated and definitively identified.

Our results indicated that UV-irradiation stimulated the synthesis of phenolic compounds in rice leaves and the majority of UV-induced phenolic compounds were phenylamides. Phenylamides are induced in response to *B. oryzae* infection and are suggested to have defense-related function [30]. *N*-*p*-Coumaroylserotonin is found both in UV-treated and *B. oryzae*-infected rice leaves. In addition, feruloyltryptamine and feruloylserotonin have been identified in *B. oryzae* infected rice leaves [30]. These findings suggest that a diverse array of phenylamides is produced in rice leaves in response to biotic and abiotic stress.

### 2.2. UV Irradiation Stimulates Phenylamide Biosynthetic Pathways in Rice Leaves

In some plant species, accumulation of phenylamides is induced by pathogens and environmental stress [22,23,24,25,26,27,28,29,30]. Analysis of salt treated- and *B. oryzae* infected-rice suggests defense-related functions of phenylamides and polyamines [29,30,34]. HPLC analysis of phenolic-enriched fractions obtained from UV-treated rice leaves showed that phenylamides are rapidly synthesized in rice leaves in response to UV irradiation and reach maximum levels about 1 or 2 days after UV treatment (Figure 1B). Similarly, accumulation of phenylamides, such as feruloyltryptamine and feruloylserotonin, in rice leaves is reached the highest levels 1 day after *B. oryzae* infection [30]. The phenolic part of phenylamides might be synthesized from phenylalanine through the phenylpropanoid pathway [31]. Transcriptome analysis of UV-treated rice leaves showed that expression of phenylpropanoid pathway genes encoding phenylalanine ammonia-lyase, cinnamate 4-hydroxylase and 4-coumarate CoA ligase is highly induced by UV irradiation [31]. These genes are most likely involved in the formation of phenolic acid-CoAs that are activated donors of an acyl group in phenylamide production [20,21]. The arylamines in UV-induced phenylamides are tryptamine and tyramine (Figure 2), which are derived from tryptophan and tyrosine, respectively. Tryptophan is synthesized from the shikimate pathway product chorismate, a branch point in aromatic amino acid biosynthesis.

**Figure 3:**
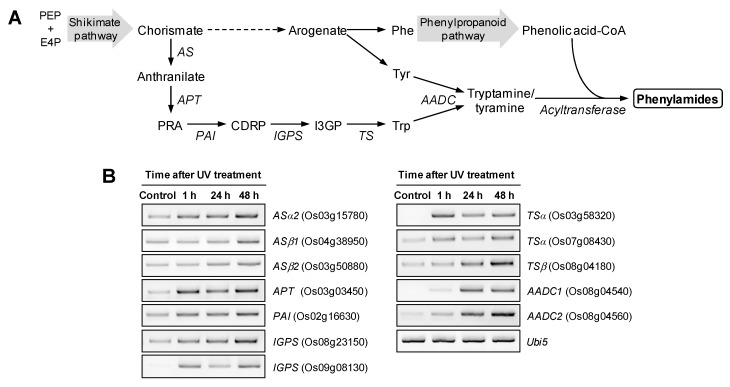
Activation of the phenylamide biosynthetic pathways in response to UV treatment. (**A**) Biosynthetic pathways in the UV-induced phenylamide production. (**B**) UV-induced expression of arylamine biosynthetic genes possibly involved in phenylamide formation. Expression of phenylamide biosynthetic genes in rice leaves 1, 24 and 48 h after UV treatment was analyzed by semi-quantitative RT-PCR. *AS*; anthranilate synthase, *APT*; anthranilate phosphoribosyltransferase, *PAI*; phosphoanthranilate isomerase, *IGPS*; indole-3-glycerol phosphate synthase, *TS*; tryptophan synthase, *AADC*; aromatic amino acid decarboxylase. PEP; phosphoenolpyruvate, E4P; erythrose 4-phosphate, PRA; phosphorubosyl anthranilate, CDRP; l-(*O*-carboxyphenylamino)-l-deoxyribulose-5-phosphate, I3GP; indole-3-glycerol phosphate.

Five enzymes are involved in tryptophan biosynthesis from chorismate (Figure 3A). Previous transcriptome analysis observed upregulation of a few genes involved in tryptophan biosynthesis in UV-treated rice leaves [31].

We analyzed the expression of genes in all steps in tryptophan biosynthesis using semi-quantitative reverse transcription polymerase change reaction (RT-PCR). The conversion of chorismate to anthranilate is catalyzed by anthranilate synthase (AS) which is composed of two subunits, ASα and ASβ (Figure 3A). Expression patterns in UV-treated rice leaves were determined for two genes for ASα (*ASα1* and *ASα2*) and three genes for ASβ (*ASβ1-3*). RT-PCR analysis showed that *OsASα2* (Os03g15780) and *OsASβ1* (Os04g38950) were upregulated in response to UV treatment and *OsASβ2* (Os03g50880) was slightly upregulated in UV-treated rice leaves (Figure 3B). Expression of *OsASα2* is reported to be induced by elicitation [35]. Upregulation of these genes was also observed in *B. oryzae-* infected rice leaves [30]. 

The next two steps from anthranilate to l-(*O*-carboxyphenylamino)-l-deoxyribulose-5-phosphate (CDRP) are catalyzed by anthranilate phosphoribosyltransferase (APT) and phosphoanthranilate isomerase (PAI) (Figure 3A). The expression of *APT* (Os03g03450) was highly induced by UV irradiation and *PAI* (Os02g16630) was slightly increased in UV-treated rice leaves (Figure 3B). Indole-3-glycerol phosphate synthase (IGPS) catalyzes the formation of indole-3-glycerol phosphate from CDRP (Figure 3A) and is important in tryptophan and auxin biosynthesis [36]. RT-PCR analysis showed that expression of two *IGPS* genes (Os08g23150 and Os09g08310) was stimulated by UV treatment (Figure 3B). The last two steps of tryptophan biosynthesis are catalyzed by tryptophan synthase (TS) which is composed of two subunits (TSα and TSβ) (Figure 3A). In the *TSα* and *TSβ* gene family, Os03g58320 and Os07g08430 for TSα and Os08g04180 for TSβ were upregulated in response to UV treatment (Figure 3B). Taken together, RT-PCR results showed that a set of genes involved in tryptophan biosynthesis was upregulated by UV treatment in rice leaves. Tyrosine biosynthesis shares biosynthetic pathways with phenylalanine, which is activated by UV treatment [31]. 

The arylamines tryptamine and tyramine are formed by decarboxylation of tryptophan and tyrosine, respectively, which is catalyzed by aromatic amino acid decarboxylase (AADC). Putative rice *AADC1* gene (Os08g04540) was expressed in *Escherichia coli* and the resulting enzyme showed tryptophan decarboxylase activity [37]. *AADC1* is upregulated in rice leaves by UV irradiation (Figure 3B). Expression of *AADC1* is increased in rice leaves by *B. oryzae* infection [30]. RT-PCR analysis of UV-treated rice leaves showed that rice *AADC2* (Os08g04560) was induced by UV irradiation (Figure 3B). This result suggested that, *AADC2* is also possibly involved in arylamine formation in UV-treated rice leaves. Phenylamide formation from arylamines and phenolic acid-CoA is likely catalyzed by acyltransferases. Previous transcriptome analysis of UV-treated rice leaves showed upregulation of several acyltransferases possibly involved in phenylamide formation [31]. Our results and previous transcriptome data suggested that arylamine and phenolic acid-CoA biosynthetic pathways are activated by UV irradiation for the phenylamide production in rice leaves.

### 2.3. UV-Induced Phenylamides in Rice Leaves are Potential Phytoalexins against Rice Pathogens

#### 2.3.1. Antimicrobial Activity of UV-Induced Phenolic Compounds

As shown in above, UV irradiation stimulated the production of phenylamides in rice leaves in addition to sakuranetin accumulation. To determine whether UV-induced phenylamides were phytoalexins, we examined the antimicrobial activity of phenylamides isolated from UV-treated rice leaves against rice pathogens. The fungal pathogens used were *M. grisea*, *R. solani*, and *B. oryzae*. To examine the antifungal activity of phenylamides and sakuranetin, the growth of fungal pathogens was measured on potato dextrose agar (PDA) plates containing different concentrations of UV-induced phenolic compounds. Sakuranetin and compound **4** inhibited the growth of the tested fungal pathogens (Table 1). The concentration of sakuranetin to inhibit 50% of mycelial growth (IC_50_) of *M. grisea* was 6.44 μg/mL. This result was comparable to the previously determined IC_50_ (5 μg/mL) of sakuranetin to germ tube growth of *M. grisea* [6]. Sakuranetin showed antifungal activity to *R. solani* and *B. oryzae* and had an IC_50_ of 54.04 μg/mL against *R. solani* and 19.05 μg/mL against *B. oryzae*. Although most phenylamides showed no significant antimicrobial activity against the tested fungal pathogens, compound **4** inhibited the growth of *B. oryzae* with an IC_50_ value of 26.92 μg/mL (Table 1).

**Table 1:** IC_50_ (μg/mL) of the phenolic compounds isolated from UV-treated rice leaves to fungal and bacterial pathogens.

Compound	Fungal Pathogen			Bacterial Pathogen		
	*M. grisea*	*R. solani*	*B. oryzae*	*Xoo*	*Xoc*	*B. glumae*
**1**	6.44 ± 1.35	54.04 ± 22.61	19.05 ± 9.83	19.95 ± 6.20	2.36 ± 0.35	8.22 ± 1.49
**2**	- ^a^	-	-	21.96 ± 7.00	3.18 ± 0.44	-
**3**	-	-	-	34.76 ± 10.98	3.72 ± 0.20	-
**4**	-	-	26.92 ± 4.74	24.34 ± 5.60	2.45 ± 0.48	41.09 ± 5.96
**5**	-	-	-	-	54.54 ± 20.84	-

^a^ No significant antimicrobial activity of the phenolic compounds to rice fungal and bacterial pathogens.

**Figure 4:**
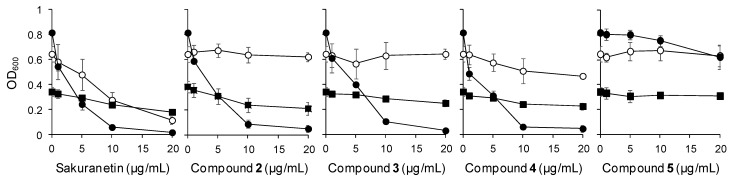
Antibacterial activity of the UV-induced phenolic compounds to bacterial pathogens, *Xoo* (■), *Xoc* (●), *B. glumae* (○).

Antimicrobial activity of the UV-induced phenolic compounds against rice bacterial pathogens was also examined using *Burkholderia glumae*, which causes bacterial grain rot; *Xanthomonas oryzae* pv. *oryzae* (*Xoo*), which causes blight; and *X. oryzae* pv. *oryzicola* (*Xoc*), which causes leaf streak. Sakuranetin and compound **4** inhibited the growth of all tested pathogens to different extents (Figure 4). 

Compounds **2** and **3** showed antimicrobial activity against *Xoo* and *Xoc* (Figure 4). Compound **5** inhibited *Xoc* growth (Figure 4). Sakuranetin and compound **4** showed the strongest inhibitory effects on *Xoc* growth with IC_50_ of 2.36 μg/mL for sakuranetin and 2.45 μg/mL for compound **4** (Table 1). Sakuranetin had an IC_50_ of 8.22 μg/mL against *B. glumae* and 19.95 μg/mL against *Xoo* (Table 1). Compound **4** similarly inhibited *Xoo* growth compared with sakuranetin and showed relatively weak antimicrobial activity to *B. glumae* (Figure 4, Table 1). Compounds **2** and **3** had slightly weaker activity than sakuranetin but inhibited *Xoc* growth with IC_50_ of 3.18 μg/mL for compound **2** and 3.72 μg/mL for compound **3** (Table 1). Compounds **2** and **3** also inhibited *Xoo* growth (Figure 4). Compound **5** showed relatively weak antimicrobial activity to *Xoc* only with an IC_50_ of 54.54 μg/mL (Figure 4, Table 1).

#### 2.3.2. UV-Induced Phenylamides Are Potential Phytoalexins in Rice 

Although phenylamides have been identified in pathogen-infected and UV-irradiated rice leaves [26,31], no studies have reported on their antimicrobial properties against rice pathogens. We examined the antifungal activity of four phenylamides and sakuranetin isolated from UV-treated rice leaves. Sakuranetin is suggested to be a strong rice phytoalexin against *M. grisea* [6]. The IC_50_ of sakuranetin against *M. grisea* germ tube growth is 5 μg/mL [6]. The IC_50_ values for rice diterpenoid phytoalexins against *M. grisea* germ tube growth are 1 to 35 μg/mL [1,3,9,12,17]. Oryzalexin D was reported to inhibit mycelial growth of *M. grisea* with an IC_50_ of 230 μg/mL [38]. Our result showed that sakuranetin strongly inhibited mycelial growth of *M. grisea* with the IC_50_ of 6.44 μg/mL (Table 1), which is comparable to its IC_50_ for the germ tube growth [6]. In addition to activity against *M. grisea*, sakuranetin inhibited *R. solani* and *B. oryzae* mycelial growth (Table 1). *R. solani* mycelial growth was reported to be inhibited by phytocassanes B and C [9]. Sakuranetin also showed antibacterial activity against all tested bacterial pathogens. In particular, sakuranetin almost completely inhibited *Xoc* growth at 10 μg/mL (Figure 4). These findings suggested that sakuranetin is a wide spectrum antimicrobial agent in rice. 

Although compound **4** showed no significant inhibition of *M. grisea*, it had antifungal activity against *B. oryzae* (Table 1). The inhibition of *B. oryzae* mycelial growth by compound **4** was comparable to inhibition by sakuranetin. Compound **4** also showed antimicrobial activity against all tested bacterial pathogens with an IC_50_ against *Xoo* and *Xoc* similar to sakuranetin (Figure 4, Table 1). These results suggested that compound **4** was a phytoalexin against a wide array of rice pathogens. Compounds **2** and **3** showed no significant inhibitory effects against fungal pathogens but strong antibacterial activity against *Xoc* comparable to sakuranetin and compound **4** (Figure 4, Table 1). Compounds **2** and **3** almost completely inhibited *Xoc* growth at 10 μg/mL (Figure 4). Compounds **2** and **3** also inhibited *Xoo* growth (Figure 4, Table 1). Compound **5** had weak antimicrobial activity against *Xoc* compared to other phenylamides and sakuranetin (Figure 4, Table 1). These results suggested that compounds **2**, **3**, and **5** were also potential phenolic phytoalexins to rice bacterial pathogens.

Phenylamides serve as phytoalexins in some plant species [20,21,25,26,27,28]. *N*-*p*-Coumaroylserotonin from bamboo has antifungal activity against *Aciculosporium take*, which cause witches’ broom disease [27]. Antimicrobial acyltyramines such as *N*-feruloyltyramine and *N*-*p*-coumaroyltyramine, which act against pathogenic bacteria and fungi, are found in gallic root and pepper leaves [25,26]. *p*-Coumaroylnoradrenaline from tomato has antimicrobial activity against the bacterial pathogen *Peudomonas syringae* pv. *tomato* [28]. Many phytoalexins have been identified from rice and their antimicrobial activity against fungal pathogens, mainly the blast fungus *M. grisea*, has been examined. However, little is known about the antimicrobial activity of phytoalexins against other rice pathogens, in particular, pathogenic bacteria. We examined the antimicrobial activity of UV-induced phenylamides and sakuranetin against rice fungal and bacterial pathogens. These phenolic compounds were antimicrobial agents against fungal pathogens and bacterial pathogens. Thus, our findings strongly suggested that, in addition to sakuranetin, UV-induced phenylamides serve as a phytoalexins in rice against a diverse array of rice pathogens.

## 3. Experimental Section

### 3.1. Plant Growth Conditions and UV Treatment

Rice plants (*Oryza sativa* L. spp *japonica* cv. *Dongjin*) were grown in a greenhouse at 28 °C during the day and 20 °C at night. Eight week-old rice plants were UV-irradiated in a growth chamber with five 20 W germicidal lamps (maximum emission at 254 nm, 7.5 W UV output, Sankyo Denki Co. Kanagawa, Japan) for 2 h. UV-treated plants were transferred to a greenhouse and leaves were collected at 1, 24, 48, and 96 h after UV treatment for phytochemical and gene expression analyses.

### 3.2. Analysis, Isolation and Identification of Phenylamides from UV-Treated Rice Leaves

Rice leaves were ground in liquid nitrogen and extracted with 70% methanol (MeOH)-water for 1 h. After centrifugation, the aqueous methanol extract was obtained and dried *in vacuo*. Residue was dissolved and fractionated in ethyl acetate (EtOAc)-water (1:1) mixture to enrich phenolic compounds. The ethyl acetate phase was dried *in vacuo* and residue was dissolved in MeOH and analyzed using a reversed phase HPLC with a Sunfire C_18_ column (4.6 × 250 mm) (Waters, Milford, MA, USA) with detection at 280 nm using elution conditions reported by Park *et al.* [32]. 

Phenolic-enriched samples from rice leaves collected 1 day after UV-treatment were applied to a silica gel column to isolate UV-induced compounds. Consecutive elution was performed with hexane–benzene (1:1), hexane–benzene (1:2), hexane–benzene (1:3), benzene–chloroform (1:1), chloroform–EtOAc (1:1), and EtOAc. Eluted fractions were analyzed by reversed phase HPLC as above Fractions containing UV-induced compounds were dried *in vacuo*. Fractions enriched in UV-induced compounds were purified by reversed phase HPLC with a preparative Sunfire C_18_ column (10 × 150 mm) (Waters) on a linear gradient of 25%–60% acetonitrile in 3% acetic acid-water for 25 min (flow rate 3 mL/min) with detection at 280 nm. 

Isolated compounds were identified using NMR spectroscopy and positive ESI-MS. NMR spectra of isolated compounds were recorded in CD_3_OD on an Avance 600 NMR spectrometer (Bruker, Rheinstetten, Germany) using tetramethylsilane as an internal standard. MS spectra were obtained with the ion source of positive mode electronspray ionization using an Agilent 6410 Triple Quadruple LC/MS System (Agilent Technologies, Santa Clara, CA, USA).

### 3.3. RNA Isolation and Semi-Quantitative RT-PCR Analysis

Total RNA was isolated from UV-treated and untreated rice leaves using TRIzol reagent (Invitrogen, Carlsbad, CA, USA) and 3 μg of RNA was used to synthesize cDNA using the RNA to cDNA EcoDry kit (Clontech, Mountain View, CA, USA) according to the manufacturer’s protocol. Semi-quantitative RT-PCR was performed with gene-specific primers. Primers and PCR conditions for each gene were summarized in Table S2. The ubiquitin gene (*Ubi5*) was the control.

### 3.4. Antimicrobial Activity

Antifungal activity of phenolic compounds from UV-treated rice leaves against three rice pathogenic fungi (*M. grisea* PO6-6, *R. solani* AG-1 and *B. oryzae*) was examined. Compounds were dissolved in dimethylsulfoxide (DMSO) and added to PDA media (Becton, Dickinson and Company, Sparks, MD, USA) at final concentrations of 1, 5, 10 and 20 µg/mL. The final DMSO concentration in media was 1% (v/v). Mycelial discs (1 mm in diameter) were cut from the edge of 2-day-old cultures of pathogens on PDA plates and placed on PDA plates containing different concentrations of compounds. Pathogenic fungi were incubated at 28 °C for three days and mycelial radius was measured to determine growth inhibition by UV-induced phenolic compounds. Experiments were performed in triplicate. 

Pathogenic bacteria (*Xoo*, *Xoc* and *B. glumae*) were grown in NB media (Becton, Dickinson and Company) at 28 °C. Cultures were inoculated in NB media containing 1, 5, 10 and 20 μg/mL compounds. The final DMSO concentration in the media was 1% (v/v). To determine inhibition by compounds, bacterial growth was examined by measuring OD_600_ during exponential growth using a V-550 UV/Vis spectrophotometer (Jasco, Tokyo, Japan). Experiments were performed in triplicate.

## 4. Conclusions 

UV irradiation stimulates the production of phytoalexins in rice leaves. Besides known phytoalexins, we isolated and identified phenylamides including *N*-*trans*-cinnamoyltryptamine and *N*-*p*-coumaroylserotonin from UV-treated rice leaves. To unravel the role of UV-induced phenylamides as phytoalexins, we examined their antimicrobial activity against rice bacterial and fungal pathogens. UV-induced phenylamide had antimicrobial activity against the tested pathogens, in particular bacterial pathogens. These results suggested that the UV-induced phenylamides are a new class of phytoalexins in rice against a diverse array of pathogens. 

## Supplementary Materials

Supplementary materials can be accessed at: http://www.mdpi.com/1420-3049/19/11/18139/s1.

## Supplementary Files

Supplementary File 1
