# Supplementary material for: Antimicrobial Activity of UV-Induced Phenylamides from Rice Leaves
Source: Molecules. 2014 Nov 6;19(11):18139–51. doi: 10.3390/molecules191118139 (PMC6271653; doi:10.3390/molecules191118139)
Supplement: Supplementary File 1 [file molecules-19-18139-s001.pdf]

Supplementary Materials

Table S1. ^{1}H (600 MHz) and ^{13}C (150 MHz) NMR chemical shifts of the isolated compounds from UV-treated rice leaves (in CD_3OD, chemical shifts in ppm).

**No.**	**4**		**5**	
	**^{1}H**	**^{13}C**	**^{1}H**	**^{13}C**
2	7.08 *s*	123.6	7.02 *s*	124.4
3		113.4		112.7
4		129.1		129.6
5	7.58 *d*	119.4	6.95 *d*	103.7
6	6.99 *dd*	119.7		151.3
7	7.07 *dd*	122.5	6.65 *dd*	112.8
8	7.32 *d*	112.4	7.15 *d*	112.5
9		138.4		133.3
10	3.01 *t*	26.5	2.92 *t*	26.6
11	3.60 *t*	41.8	3.56 *t*	41.6
1'		136.5		127.9
2'	7.53 *d*	129.0	7.39 *d*	130.7
3'	7.37 *m*	130.1	6.78 *d*	116.9
4'	7.37 *m*	130.9		160.7
5'	7.37 *m*	130.1	6.78 *d*	116.9
6'	7.53 *d*	129.0	7.39 *d*	130.7
7'	7.52 *d*	141.7	7.44 *d*	141.9
8'	6.58 *d*	122.2	6.39 *d*	118.7
9'		168.8		169.4

Table S2. Sequence of gene specific primers used for semi-quantitative RT-PCR and PCR conditions.

Gene	Primer Sequence	Annealing Temperature (°C)	Cycle No.
ASα2 (Os03g15780)	5'-AATCTTGATCGGCATGCAAC-3'<br>5'-CAGCCTCTTGCCTGTACATA-3'	56	29
ASβ1 (Os04g38950)	5'-TGCAGACTGTTCTGGAACTT-3'<br>5'-TAAGGCCATCTTCAGTCCAC-3'	56	29
ASβ2 (Os03g50880)	5'-GGGAGCTAACTTTGAGGTGT-3'<br>5'-GATCCTTTCCCATGCACAAC-3'	56	29
APT (Os03g03450)	5'-GACCTACGAAGAGATCGTGG-3'<br>5'-AATCCAACACCCACCTCATT-3'	56	29
PAI (Os02g16630)	5'-TCAGTTGGTGTCTTTGTGGA-3'<br>5'-TTGAATCCCTTGCCACTTCC-3	56	29
IGPS (Os08g23150)	5'-CGGAAGTCAGGAGTGAAGTG-3'<br>5'-TTCCCTTCTCCCTGATGGTA-3'	56	29
IGPS (Os09g08130)	5'-ATCTGCAAGAACCTCGGAAT-3'<br>5'-CCAACTTAGCTGAAAACGGG-3'	52	33
TSα (Os03g58320)	5'-TTCCTTACGTAGAGACATCCAC-3'<br>5'-CAATGATCACTCCATCTGCAC-3'	56	29
TSα (Os07g08430)	5'-TAAAAGAAGCCGGTGTCCAT-3'<br>5'-CGTGACCTGCTTGATATCCT-3'	56	29
TSβ (Os08g04180)	5'-ACATGGGTGCTCAGGATATG-3'<br>5'-CAAGCAACCAAGACATCAGG-3'	56	29
AADC1 (Os08g04540)	5'-TGGACTTCATCTCGGACTAC-3'<br>5'-GTCCCCAGAATAACCCCAC-3'	52	33
AADC2 (Os08g04560)	5'-CGTGTCGACTCCATCAGCA-3'<br>5'-GACGAGGTCCTCGAACGT-3'	56	29
Ubi5 (Os01g22490)	5'-GACTACAACATCCAGAAGGAGTC-3'<br>5'-TCATCTAATAACCAGTTCGATTTC-3'	54	25
